# The Multifaceted Role of Osteopontin in Modulating the Tumor Microenvironment

**DOI:** 10.1158/0008-5472.CAN-25-1486

**Published:** 2025-08-27

**Authors:** Yu Gu, William J. Muller

**Affiliations:** 1 Rosalind and Morris Goodman Cancer Institute, McGill University, Montreal, Canada.; 2 Faculty of Medicine and Health Sciences, Department of Biochemistry, McGill University, Montreal, Canada.; 3 Faculty of Medicine, McGill University, Montreal, Canada.

## Abstract

Osteopontin (OPN), a key structural protein in the extracellular matrix, plays a pivotal role in regulating the local tumor microenvironment and systemic immunity during cancer progression. Recognizing that tumor cells do not exist in isolation but rather interact with a multitude of stromal components that significantly influence patient outcomes and therapy responses, this review focuses on the role of OPN in nontumor cells in the context of solid cancers and the associated phenotypic and mechanistic insights. We explore how OPN influences the behavior of various innate and adaptive immune cells, including NK cells, neutrophils, macrophages, dendritic cells, myeloid-derived suppressor cells, B cells, and T cells, as well as structural stromal cells such as fibroblasts, endothelial cells, and adipocytes. The review highlights the roles of OPN in modulating these stromal cells and their multiaxial influence on tumorigenesis and metastasis. These complex interplays offer insights into potential diagnostic and therapeutic strategies around OPN/stromal-mediated pathways in cancer progression and recurrence.

## Osteopontin at a Glance

Osteopontin (OPN) was initially discovered as a bone matrix protein secreted by osteoblasts and named secreted phosphoprotein 1 (Spp1), hence its gene name *Spp1* (1). *Spp1* is comprised of seven exons in mammals with five possible isoforms, the sequences and structures of which have been well summarized previously (2). Briefly, OPN-a, OPN-b, OPN-c, OPN-4, and OPN-5 isoforms have all been documented in different pathologic conditions (3, 4).

Notably, OPN’s versatility in cellular functions arises from its role as a well-defined extracellular matrix (ECM) protein with extensive posttranslational modifications that extend its structural and functional range, including glycosylation, phosphorylation, tyrosine sulfation, and sialylation sites, all of which contribute to its size range of 25 to 75 kDa (5, 6). These posttranslational modifications and proteolytic cleavages by thrombin and matrix metalloproteinases (MMP) lead to different exposures of OPN’s receptor-binding sites, both arginine–glycine–aspartic acid (RGD)–containing and non–RGD-containing domains, thus determining which receptor it preferentially binds to (7–9). Cell surface receptor CD44 and integrin heterodimers with αV, α5, α4, α9, β1, β3, β5, β6, and β8 subunits are known canonical OPN receptors, each inducing a distinct signal transduction pathway, such as PI3K/AKT, NF-κB, and ERK/MAPK, that mediates a range of cellular functions like motility, survival, and inflammation (3, 9, 10). Conversely, numerous transcription factors are known to regulate OPN expression, including Stat3, NF-κB, c-Jun, and T-bet, as well as epidermal, platelet-derived, and transforming growth factors (11–14).

OPN is constitutively expressed in many cell types and tissues but is often upregulated when tissue homeostasis is disrupted, such as damage, inflammation, and malignancy (9). Throughout the stepwise progression of solid cancers, OPN contributes to each stage of disease development, from stimulating primary tumor cell growth to angiogenesis, tumor microenvironment (TME) remodeling through fibrosis, modulating immune and stromal cell populations, and in the metastatic cascade (9, 15, 16). Additionally, functional OPN protein can remain in the cell cytoplasm, where it contributes to diverse cell functions such as structure, motility, and cell cycle as intracellular OPN (17).

**Figure 1.**
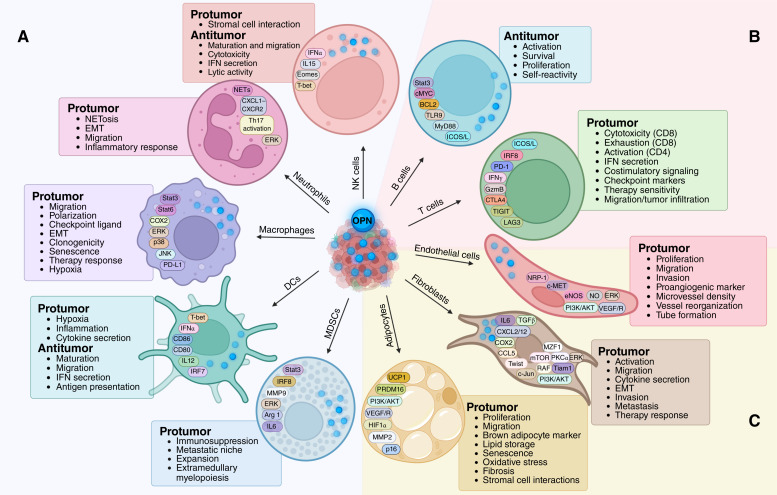
Schematic summary of the cellular functions and signaling pathways mediated by OPN on tumor stromal cells. **A,** Tumor cell–derived OPN engages with its receptors on innate immune cells, including NK cells, neutrophils, macrophages, DCs, and MDSCs, to activate signaling pathways that mediate cell motility, cytokine and IFN secretion, cellular toxicity, and cell-specific functions such as clonogenicity and senescence in macrophages and extramedullary myelopoiesis in MDSCs. Simultaneously, some innate immune cells produce intracellular OPN that further modulates these pathways and associated cellular functions. Although most phenotypes support tumorigenesis, metastasis, and therapy resistance, OPN can elicit antitumor responses in NK and DCs. **B,** Although OPN affects antigen presentation and T-cell checkpoint ligand expression on innate immune cells, bridging to the adaptive immune system, its direct role on B-cell and T-cell functions significantly affects tumor immunity and response to immunotherapies. Environmental and B-cell–derived OPN ensures its activation and survival, which confers tumor-protective roles. Conversely, OPN dampens T-cell function and modulates its checkpoint receptor expression, supporting immune evasion from T-cell–mediated tumor clearance. **C,** Tumor-infiltrated endothelial cells, fibroblasts, and adipocytes are widely affected by OPN, which can shift vessel structures, tumor matrices, and tumor metabolism to reshape the acellular TME, respectively. Between the activation of several signaling cascades, such as ERK and AKT, environmental and intracellular OPN favor tumor growth, metastasis, angiogenesis, and therapy response through these stromal cells. Of note, the effects of OPN on these stromal cells are not restricted to its cell population but often contribute to a paracrine feedback between stromal cells and tumor cells and between different stromal cell populations within a tumor. The net effect on tumorigenesis of the cellular functions mediated by OPN is indicated for each cell type. NETosis, NET release; UCP1, uncoupling protein 1. Created in BioRender. Muller, W. (2025) https://BioRender.com/vm6ck5y.

Although abundant studies and reviews explore the dysregulation and functions of OPN in cancer, there remains a gap in synthesizing its influence on the diverse tumor stromal cell populations. In this review, we summarize the current literature on OPN’s impact on innate and adaptive immune cells, fibroblasts, endothelial cells, and adipocytes across solid cancers, including mechanisms and phenotypes across various preclinical models and in clinical contexts (Fig. 1). We further expand on these discussions for future directions in OPN-based research in oncology, aiming to best leverage it for biomarker and therapeutic purposes.

## NK Cells

Although most cancer immunotherapies focus on boosting the cytotoxic activity of T cells, a vast population of innate immune cells harbors exceptional yet unharvested antitumor potential. Among them are the NK cells, which possess similar cytotoxicity to CD8^+^ T cells and are capable of tumor cell clearance (18–20). One of the benefits of NK cell–mediated tumor killing stems from taking advantage of the lack of MHC I expression on tumor cells, rendering them targets of NK cells (18). In addition to their intrinsic cytotoxicity, NK cells can also abundantly secrete cytokines and initiate the relay to adaptive immunity (21). Tissue-resident NK cells can collaborate with other stromal cells to mutually enhance each other’s antitumor function and acquire phenotypes like T cells in the same vicinity. Indeed, it has been reported that these tissue-resident NK cells can respond to anti–PD-1 and anti–CTLA4 immunotherapies (22). With more patients becoming resistant to T-cell–based immune checkpoint blockades, shifting the spotlight to NK cells with potent antitumor innate immunity could have exponential benefits. Nevertheless, given that NK cells are only beginning to rise in cancer immunotherapies, limited studies have explored the relationship between OPN and NK cells.

The majority of studies concur with the protumorigenic role of OPN in cancer progression and metastasis (9, 15). However, some showed contrary results when examining the role of OPN on NK cells. In an *in vivo* model of melanoma, full-body OPN-deficient mice failed to mount sufficient NK-cell activity against tumor growth and metastasis (12). By contrast, wild-type mice were able to elicit an IFNα-dependent cytotoxic NK-cell response (12). Soon after, it was reported that NK cell–intrinsic OPN is necessary for proper NK-cell development and function. Not only did OPN-deficient NK cells undergo more apoptosis, but also the surviving cells had noticeable impairment in maturation and were desensitized to IL15 (23). OPN-deficient NK cells were unable to limit melanoma lung metastases *in vivo* and displayed a significant reduction in their lytic activity and cytokine production (12). Mechanistically, it was proposed that the disruption in Eomes and T-bet activities from OPN deficiency contributed to the disruption of homeostasis, maturation, and function in NK cells (24).

Lung cancer consistently dominates as the top contributor to cancer-related deaths worldwide (25, 26). Eighty-five percent of all lung cancer cases are non–small cell lung cancers, which are further divided into two main subtypes: lung adenocarcinoma and lung squamous carcinoma (LUSC; ref. 27). Both subtypes can present significant variations in their genomic and TME landscapes. Single-cell RNA sequencing on patient samples revealed that a *Spp1*^high^ macrophage population enriched in LUSC showed greater interaction with NK cells (27). In turn, NK cells in LUSC themselves also expressed higher *Spp1* than did NK cells in lung adenocarcinoma (27).

Comparatively, in a separate study of lung cancer, Zheng and colleagues (28) found that lung adenocarcinoma tumors with high OPN in the TME have fewer NK cells, especially active NK cells. Similar findings were also reported in mice with prostate tumors, in which OPN-deficient mice exhibited faster tumor growth and size (29). Further analyses revealed that OPN-deficient tumor-bearing mice had decreased leukocyte infiltration, notably NK cells and dendritic cells (DC; ref. 29). This was supported by the decreased cell migration in NK cells isolated from OPN-deficient tumor-bearing mice compared with wild-type tumor-bearing mice (29). These studies demonstrate that environmental OPN influences NK-cell recruitment and activation. Therefore, environmental OPN and NK cell–intrinsic OPN could have opposing effects within a tumor, which argues the importance of examining the source, the posttranslational modifications, and the localization of intratumor OPN.

Collectively, these findings support dual roles of OPN in modulating NK-cell activity within the TME, both intracellularly and under paracrine influence. The effects of NK cell–intrinsic OPN for its adequate antitumor function and the environmental OPN’s role on NK-cell recruitment into the TME argue that further investigations are needed to decipher if and how OPN can serve as a faithful validation checkpoint and be optimized into NK cell–based immunotherapy.

## Neutrophils

Neutrophils are the most abundant immune cell type in the human body and the first line of defense in innate immunity to counter any disruption in tissue homeostasis (30). Like most tumor stromal cells, neutrophils can play dual roles on the anti- to protumorigenic spectrum. Neutrophils can differentiate to enhance antitumor immunity directly through cytotoxicity with nitric oxide production or indirectly by antigen presentation to elicit adaptive immune cells. Conversely, neutrophils can give in to the chronic influence of tumors and support tumor growth, angiogenesis, metastasis, and immune evasion. The complexity of neutrophil-dependent functions is extensively reviewed elsewhere (30, 31).

As an ECM protein, it is well established that OPN can stimulate cell migration, including for neutrophils, in an RGD-dependent fashion, wherein high OPN tumor neighborhoods have increased neutrophil infiltration (32). In a pan-cancer study, Shen and colleagues (33) delineated a neutrophil extracellular trap (NET) signature profile associated with inflammation, metastasis, and poor patient outcomes, among which, *Spp1* emerged as one of the top NET-related genes contributing to malignancies. Furthermore, the results showed an additive phenotype between *Spp1* and NET formation, in which *Spp1*^high^ NET^high^ cancers have significantly increased epithelial-to-mesenchymal transition (EMT) scores, suggesting that OPN promotes invasion through NETs in the metastatic cascade (33). Indeed, *in vitro* validation confirmed that neutrophil chemotaxis and NET formation are induced by exogenous OPN (33). This elucidates a protumorigenic relationship between OPN and NETs and is a predictor of patient survival.

In hepatocellular carcinoma (HCC), OPN is mainly sourced from malignant epithelial cells, and high levels of *Spp1* correlated with decreased overall survival (OS) and lung metastasis (34). OPN promoted lung colonization of HCC by binding to lung epithelial CD44 to induce CXCL1 secretion driven by Stat3 (34). In turn, neutrophils were recruited to the lung metastatic niche through the CXCL1–CXCR2 binding axis, wherein they were subsequently activated by OPN to produce NETs after ERK activation (34). These NETs conferred a protective role for disseminating cancer cells in the lung metastatic niche. Targeting the CXCL1–CXCR2 axis, anti-OPN, anti-Ly6G, and DNase I treatments phenocopied each other in reducing HCC lung metastatic incidences *in vivo* (34). Moreover, in a synthetic metastatic niche model of breast cancer, *Spp1* emerged as a niche-specific gene signature for neutrophils and was associated with a Th17 response–dependent recruitment and activation (35).

The pivotal roles of OPN in orchestrating prometastatic processes through neutrophils extend from recruitment to activation and NET formation. Given the multifaceted roles of OPN in establishing a favorable soil for metastasis and that plasma OPN correlated with metastasis in patients with cancer, OPN should be leveraged as a biomarker of metastasis and recurrence for early preventative treatment, especially in tandem with early immune responders like neutrophils (34). It remains to be seen if the abundance and localization of OPN vary with NET formation at different stages in the metastatic cascade and if circulating OPN-driven NETs confer a protective role for circulating tumor cells that can be clinically exploited.

## Macrophages

The involvement of tumor-associated macrophages (TAM) in cancer progression highlights the potential of cellular plasticity. These innate immune cells function to ensure tissue homeostasis through phagocytosis, antigen presentation, tissue remodeling, cytokine secretion, and inflammation modulation (36–39). In response to polarization cues within solid tumors, TAMs acquire different characteristics of either a pro- or antitumorigenic state, the spectrum of which is well defined (40–42). Polarized macrophages can directly affect cancer cells by secreting a unique set of cytokines and by restructuring the TME. Both TAM-derived OPN and OPN sourced from cancer and other stromal cells can influence their functions and tumor progression.

In several solid cancers, tumor environmental OPN has been shown to recruit and skew macrophages to a protumorigenic state correlated with accelerated tumorigenesis and worse outcomes (43, 44). It was reported in glioblastoma that both tumor- and host-derived OPN maintain the recruitment and the protumorigenicity of αvβ5 integrin+ macrophages while restricting T-cell–mediated tumor clearance (44). Melanoma isografts displayed reduced tumor growth and macrophage infiltration in OPN-deficient mice, suggesting that OPN within the TME aids in recruiting TAMs to support tumor development (45). Furthermore, OPN increased COX2 expression, ERK1/2 activation, and p38 in macrophages, which in turn stimulated endothelial cell migration and angiogenesis *in vitro* (45). Tumor cell–derived OPN has been shown to recruit macrophages into recurrent breast tumors, in which they are then synergistically polarized by IL4 and OPN (46). These protumorigenic macrophages promoted recurrence by stimulating neighboring cancer cell proliferation and tissue fibrosis, and anti-OPN treatment and macrophage depletion comparatively reduced tumor burden *in vivo* (46).

Macrophages can also express OPN and influence tumorigenesis. For example, OPN^+^ CD204^+^ macrophages predicted decreased survival in patients with gastric cancer and were associated with higher tumor stage and lymph node metastasis (47). In colorectal cancer, not only is OPN/*Spp1* expression higher in malignant tissue but is also associated with poor survival and metastasis through EMT (48). Specifically, in liver metastases of patients with poor-outcome colorectal cancer, *Spp1* was significantly higher and enriched in TAM populations with EMT signatures (49). The reciprocal paracrine relationship between OPN-expressing TAMs and tumor cells extends from senescence to clonogenicity. *Spp1*+ TAMs exhibited senescence-associated secretory phenotype, which were spatially located in closer proximity to adjacent senescent cancer cells in high-grade colon tumors (50). Human monocytes THP-1 cocultured with colorectal cancer cells secrete more OPN only when the latter expresses the CD44 receptor (51). Given the roles of CD44 in stemness, TAM-derived OPN does stimulate clonogenicity through activation of the JNK pathway (51).

Several studies in lung cancer used OPN/*Spp1* as a marker for TAM subpopulation classification (27, 52, 53). OPN/*Spp1* expression emerged as one of the markers in a hypoxic macrophage transcriptome, a predictor of worse outcomes in breast cancer (54). *Spp1*+ expression in lung carcinoma TAMs that coexpress other protumorigenic markers correlated with a worse overall clinical outcome (55). Furthermore, OPN secreted from macrophages suppressed cancer cell apoptosis and conferred resistance to chemotherapies (55). Reciprocally, OPN secreted by lung cancer cells induced macrophage polarization into acquiring CD206 and arginase 1 expression (55). Additionally, inhibition of *Spp1* in macrophages decreased their PD-L1 expression, dampened their protumorigenic phenotype, and elevated their T-cell activation capacity (56). Overall, targeting OPN in lung cancer TAMs proved to be advantageous for both chemotherapy and immunotherapy. Therefore, macrophages’ intrinsic expression of OPN could be a precursor to its polarization status from external stimuli and a determinant in PD-L1 expression (56, 57). TAM-derived OPN was further validated as an independent prognostic factor of both OS and disease-free survival (53). Thus, OPN+ TAMs are not only a significant determinant in patient outcomes but also in immunotherapy response.

Overall, OPN plays a protumorigenic role on TAMs, such as their recruitment and polarization. These OPN-induced shifts in TAMs often elicit a secondary line of protumorigenic waves like EMT, angiogenesis, immunosuppression, and fibrosis. Furthermore, OPN serves as a marker for TAM subtypes that are associated with poor clinical outcomes, including evidence of suppressing T-cell activity. Although several groups investigated the potential of targeting OPN in or with TAMs with promising results, few investigations have done so while distinguishing circulating macrophages and tissue-resident macrophages, particularly specialized macrophages such as microglia that may be highly pertinent for rare cancers and associated pathologic conditions.

## DCs

DCs are descendants of monocyte–DC progenitors and are one of the main antigen-presenting cells (APC; refs. 58, 59). DCs’ roles as the bridge between innate and adaptive immunities are well established in infection and inflammation (60–62). Specifically, upon recognition of pathogen and tissue-damaged patterns, DCs elicit regulatory and helper T-cell responses (63). In recent years, an increasing number of studies have been dedicated to understanding and exploiting DC functions in cancer development and cancer treatments, which are reviewed elsewhere (58). Secreted and intracellular OPN regulate DC survival, maturation, migration, antigen presentation, and cytokine secretion to ensure adequate antipathogenic immunity (63). However, limited studies have specifically investigated the role of OPN on DCs in cancer. Some notable results highlighted below carry translational potential into cancer immunology beyond the migratory impact of OPN (43).

The majority of HCC cases are associated with chronic liver disease, including viral hepatitis (64). DC functions are often disrupted in patients with chronic hepatitis B (CHB), wherein they have decreased OPN secretion compared with their healthy counterparts (65, 66). Notably, OPN was crucial in the proper development and maturation of DCs, in which OPN inhibition decreased CD80, CD86, and IL12 costimulatory molecules after HBV antigen stimulation (65). Consequently, these low OPN-expressing DCs failed to mount a sufficient Th1 response and present HBV antigens (65). OPN supplementation significantly improved the function of DCs isolated from patients with OPN^low^ CHB (65). Altogether, these results suggest that OPN plays an important role in DC-mediated HBV response with the potential to prevent patients with CHB from progressing toward liver cancer.

One mechanism of OPN-dependent antigen presentation in plasmacytoid DCs was revealed to be dependent on transcription factor T-bet, in which intracellular OPN drives the expression of IFNα through association with MyD88 and IRF7 (67). Interestingly, this pathway triggered by OPN is independent of NF-κB and its downstream cytokines. OPN-deficient plasmacytoid DCs could not present antigens as effectively, with a specific defect in antigen uptake mediated by IFNα, an important mediator of immune response in cancer with therapeutic implications (12, 68).

Inadequate vascularization in solid tumors is a well-documented contributor to hypoxia, which can restrict nutrient availability and interfere with adequate drug delivery and distribution (69). DCs are particularly sensitive to hypoxic conditions, which can alter the expression profile of human monocyte–derived mature DCs (mDC; refs. 70, 71). This shift reflected increases in inflammatory and angiogenic chemokines and cytokines in mDCs, known to affect a secondary line of immune cell functions such as neutrophil recruitment and Th-cell activation (70). Within the list, OPN was revealed to be increased more than sixfold in mDCs along with a parallel increase in secreted OPN in conditioned media of hypoxic mDCs (70). Therefore, it is critical to evaluate stromal cell populations within hypoxic tumor regions as their response to hypoxia may alter tumor outcomes.

The immunogenic potential and plasticity of DCs remain active fields of investigation, particularly surrounding ECMs such as OPN. Given their advantageous position between the innate and adaptive immunities and the rise of cancer and DC-based vaccines, the cell-intrinsic and cell-extrinsic impacts of OPN in late-stage malignancies are curious research crossroads. By extension, the antitumor function and influence on DC-mediated T-cell activation and subsequent effect on immunotherapy also hold valuable clinical implications to be explored.

## Myeloid-Derived Suppressor Cells

In the cancer immunoediting model, entry and sustenance of the immune escape phase allow for successful tumor progression (72, 73). The main adaptation that permits a fruitful escape from immune surveillance is the effective suppression of the host’s immune system. Several tumor stromal cells take on this role, including myeloid-derived suppressor cells (MDSC; refs. 73, 74). MDSCs can phenotypically resemble monocytes or polymorphonuclear neutrophils, each with a distinct gene expression profile and cell surface markers to effectively support hallmarks of cancer (75). In many solid cancers, the number of MDSCs within the tumor can be low, with the majority residing in peripheral organs such as the spleen or the bone marrow (76, 77). Their shift into a protumorigenic and immunosuppressive state at those distal sites could be critical in establishing a favorable tumor “macroenvironment” and premetastatic niche to facilitate systemic immunosuppression and metastasis (78, 79). One of the transcription factors that promotes MDSC expansion is Stat3, with an indirect influence on their mobilization and survival (80). As a Stat3 target, OPN inevitably contributes to this process, further extending its protumorigenic role through MDSCs (14, 46).

In an *in vivo* model of metastatic breast cancer, host MDSC-derived OPN was shown to help establish an immunosuppressive metastatic niche. Notably, monocytic MDSCs in the lungs of breast tumor–bearing mice expressed a significant amount of OPN, with most being retained in the cell cytoplasm (81). In OPN knockout mice, monocytic MDSCs in the lungs displayed fewer immunosuppressive phenotypes, including a decrease in Stat3, IL6, and arginase 1 expression, resulting in a corresponding decrease in lung metastasis and T-cell infiltration (81). In primary tumor-bearing mice, the lack of OPN decreased a granulocytic subpopulation of Gr-1^low^ immunosuppressive MDSCs when compared with tumor-free mice (81). Mechanistically, it has been proposed that myeloid cells without the transcription factor IRF8 upregulated OPN expression and consequently suppressed T-cell activity (82). Therefore, OPN, directly and indirectly, contributes to immune escape for successful tumor progression and metastasis by priming an immunosuppressive metastatic niche through MDSCs and, to an extent, T-cell activity (81, 83).

The differentiation and expansion of MDSCs from hematopoietic stem cells normally occur in the bone marrow (76, 77). However, under pathologic conditions like cancer, this process can happen elsewhere, termed extramedullary myelopoiesis (84, 85). Given the protumorigenic roles of MDSC in cancer progression, extramedullary myelopoiesis can abnormally increase MDSC numbers and thus confer a macro-immunosuppressive environment. In a mouse model of colorectal cancer, tumor-derived OPN not only increased myeloid progenitor cell proliferation but also splenic extramedullary myelopoiesis (84). This phenotype was the result of ERK/MAPK activity downstream of CD44 activation upon OPN stimulation (84).

MDSCs were documented to accumulate in the spleens of lung tumor–bearing mice (86). Lung cancer cell–conditioned media were able to expand MDSC populations, with OPN and MMP9 predominantly found in the extracts (87). Considering that OPN has numerous MMP enzymatic cleavage sites, the increased MMP9 levels with increased OPN suggested that a specific cleaved-OPN isoform may contribute to MDSC expansion. Indeed, it was found that an MMP9-cleaved 32-kDa OPN isoform was responsible for MDSC expansion (87).

Altogether, these studies demonstrate that OPN can exert a significant effect on MDSC expansion, recruitment, and immunosuppressive activities that bridge to the adaptive immunity in the context of cancer. In perspective, the relative contribution between exogenous tumor-secreted OPN and MDSC intracellular OPN for these phenotypes remains to be elucidated with consideration for OPN isoforms and variants in activating different signaling pathways in MDSCs (88). Particularly, the capability of MDSCs in influencing metastatic niches through systemic immunosuppression under the influence of OPN presents a unique and advantageous field of study that can significantly improve clinical prevention in metastasis and recurrence.

## B Cells

Shortly after the expansion of T-cell–based immunotherapies in cancer, the field observed an influx of studies on B cells and plasma cells. B cells are the main APCs and antibody-secreting cells, the roles of which in cancer are well examined (89–91). Briefly, tumor-infiltrating B cells can enhance antitumor immunity by activating CD8^+^ T cells and producing tumor-specific antibodies (89–91). However, certain subsets of B cells, like regulatory B cells, can suppress immune responses and promote tumor growth by secreting immunosuppressive cytokines (92, 93).

Although direct investigations on the cross-talk between OPN and B cells in solid cancers are relatively rare, some studies have investigated this in autoimmune diseases that predispose patients to latent cancers (94). Namely, systemic lupus is associated with hematologic malignancies and lymphoma risks, in which OPN deficiency accelerated tumor incidence with B cells expressing BCL2, c-MYC, and activated Stat3 signaling (95). Therefore, OPN acts as a repressor of the Stat3 pathways specifically in B cells and may protect against malignancies derived from autoimmune diseases (95). Furthermore, OPN has been shown to play a role in Sjögren syndrome, an autoimmune disease wherein patients are at a higher risk of developing non–Hodgkin B-cell lymphoma (96). Specifically, B cell–derived OPN helped B-cell survival and expansion, contributing to self-reactivity and worsening of Sjögren syndrome, increasing the risks of B-cell lymphoma (97). In addition to OPN’s binding to its canonical CD44 and integrin receptors, a thrombin-cleaved OPN isoform was found to be able to bind to the costimulatory molecule inducible T-cell costimulator ligand (ICOSL), which could affect tumor-infiltrating B cell function as ICOSL expression on antigen-presenting B cells has been well documented (98–100).

Although there are currently limited studies directly examining the relationship between OPN and B cells in solid cancers, evidence from autoimmune disease models suggests that OPN can mediate various B-cell functions. These findings point to a relatively context-dependent relationship that would impact cancer risk and progression. Given the increasing focus on tumor-infiltrating B cells in cancer immunology, future research should explore the functional consequences of OPN signaling in B cells. Clinically, this could open new avenues targeting the OPN–B cell axis, particularly for patients with lymphoma and those with an autoimmune background.

## T Cells

T lymphocytes have long been the gravitational point in cancer immunology. From the discovery of PD-1/PD-L1 and CTLA4 to the commercialization of immune checkpoint inhibitors and, more recently, optimizing chimeric antigen receptor T-cell therapies, the versatility of T cells in tumors offers a unique diving board for the development of cancer immunotherapies (101–104). Interestingly, in 1989, Patarca and colleagues (105) independently mapped an early T-lymphocyte activation 1 (ETA-1) protein with high sequence homology to OPN, which was discovered just 3 years earlier in rat bones (106). ETA-1 (now called OPN) was then described to have an impact in the early stages of bacterial infection, playing a critical role in the early phases of cell-mediated immune response (107). Since then, several groups have investigated the role of OPN on T-cell activity in the context of cancer, with findings that provide exciting avenues and strategies that can optimize current T-cell–based immunotherapies. Intrinsically, OPN expression and secretion by T cells are relatively low with limited colocalization of CD4^+^ T lymphocytes and OPN in adult T-cell leukemia (108).

In colon cancer, IRF8 in epithelial and myeloid regulatory cells was identified as a repressor of OPN, which in turn dampens cytotoxic CD8^+^ T-cell response. *Spp1* contains two IFN-stimulated response elements in which IRF8 binds to repress OPN expression (82). In IRF8-knockout mice, CD44^high^ CD8^+^ T-cell population increased along with a 10-fold increase in OPN. The high levels of OPN in turn inhibit CD8^+^ T-cell activation and IFNγ secretion (82). Similar results were also observed in breast cancer and renal cell carcinoma (46, 109). Altogether, tumor-derived OPN and its engagement with CD44 on CD8^+^ T cells present an additional checkpoint that dampens T-cell activation, and perhaps an exploitable vulnerability, that confers immunotherapy resistance (82).

Three years after these findings, Klement and colleagues (110) subsequently identified another OPN-mediated mechanism of anti–PD-1 resistance. Systemic OPN levels were higher in tumor-bearing mice, and the deletion of OPN in colon carcinoma epithelial cells decreased tumor growth *in vivo* with an increase in cytotoxic T-cell activity. Specifically, neutralizing OPN allowed these cytotoxic T cells to acquire enhanced lytic activity and effectively decreased tumor burden in mice to a comparable degree as anti–PD-1 (110). Similarly, in a transgenic mouse model of breast cancer, anti–OPN-treated mice displayed T-cell–mediated tumor clearance (46). Targeting OPN resensitized resistant mammary tumors to anti–PD-1 by increasing granzyme B+ active T-cell infiltration, resulting in decreased tumor burden (46). In addition to modulating PD-1, OPN has also been shown to activate PD-L1 expression on macrophages in lung cancer, influencing T-cell activity (56). These promising results offer an alternative therapeutic avenue for patients with cancer who are intrinsically nonresponders or develop resistance to PD-1/PD-L1–based immune checkpoint inhibitors (110).

As mentioned previously, a subset of OPN isoforms can engage with ICOSL on APCs, which provides costimulatory signaling to its receptor ICOS on the surface of activated T-cells (98–100). In the study, two ICOSL extracellular domain-interacting sites were found at each terminus of OPN, both corresponding to the biological cleavage of OPN by thrombin (100). Given this newly found interaction, it is likely that OPN^high^ tumors have dampened T-cell activation because of decreased ICOS–ICOSL interaction, in which OPN acts as a binding partner on ICOSL (100). Indeed, ICOS expression was required to promote regulatory T-cell expansion, in which OPN competitively interacts with ICOSL in melanoma TMEs, a phenomenon especially prominent in metastases (111). Further investigations are required to validate and expand these findings and their clinical implications in conventional immunotherapies.

Ovarian cancer continues to lead as the deadliest gynecologic cancer, with most patients being subpar candidates for immunotherapy (112–114). OPN was found to be elevated in ovarian tumor tissues compared with normal tissues and unsurprisingly correlated with poor patient outcomes (43, 115–117). Notably, OPN positively correlated with increased immune cell infiltration in ovarian cancer, exerting its effect globally on most immune cell types (43). Among them, CD8^+^ T-cell expression was higher in OPN^high^ patient samples (43). Yet, this was in tandem with an increase in numerous immune checkpoint markers: PD-L1, CTLA4, LAG3, and TIGIT (43). Therefore, in addition to its recruitment capabilities, OPN also contributes to T-cell exhaustion (43).

Modulating T-cell activity opened the doors for the field of cancer immunotherapies in the last few decades with incredible success. Yet, like most targeted therapies, tumor screening remains the bottleneck for optimal response, expanding from the number of tumor-infiltrating lymphocytes to their spatial distribution and activity status. In this study, preclinical data suggest that OPN may be an additional checkpoint to be considered when evaluating anti–PD-1 effectiveness. Additionally, the indirect effects of OPN on T cells through other stromal cells must be taken into consideration, warranting further *in vivo* studies with immunocompetent preclinical models. Furthermore, it is worthwhile to evaluate the effectiveness of targeting OPN to optimize anti–PD-1 response stratified by tumor stiffness, especially for tumors prone to fibrosis, to better understand the biomechanical implications in advancing immunotherapies.

## Fibroblasts

Matrix homeostasis ensures tissue integrity and proper wound healing (118, 119). Fibrosis occurs when this balance is disturbed, leading to the excessive accumulation of connective tissue. In solid tumors, ECM dysregulation contributes to desmoplasia, which has been shown to provide both anti- and protumorigenic cues (120–123). Fibroblasts are descendants of mesenchymal lineage, are the major source of ECMs and MMPs, and are mainly responsible for the regulation of the acellular tissue integrity (124). Once activated into cancer-associated fibroblasts (CAF) in tumors, they acquire distinct autocrine and paracrine signaling characteristics. Recently, the bifurcated activation of fibroblasts into CAFs has been reformatted into a spectrum, in which CAFs can be pro- or antitumorigenic, like most immune stromal cells (125–128).

Numerous studies investigated the intricate relationship between OPN and CAFs, most concurring with a protumorigenic association. Phenotypically, α9β1 integrin engagement on MDA-MB-231 breast cancer cell line significantly promoted tumor growth and CAF recruitment (129). Reciprocally, CAFs stimulated OPN secretion by cancer cells and tumor growth (129). Consistently, tumor growth was only enhanced by cotransplanting MDA-MB-231 breast cancer cells with OPN-expressing mouse embryonic fibroblasts but not OPN-deficient mouse embryonic fibroblasts, supporting a synergistic positive loop between cancer cell–derived and CAF-derived OPN (129). Similarly, OPN in mouse mammary tumor virus promoter–polyomavirus middle T antigen–derived breast cancer cell–conditioned media was able to activate primary fibroblasts to a proinflammatory, CAF-like state through αvβ3 integrin and CD44 receptors (130). Interestingly, blockage of each OPN receptor alone dampened a different set of cytokines: for example, CXCL2 secretion was only decreased upon CD44 inhibition but not αvβ3 integrin inhibition, whereas dampening COX2 required dual inhibition of αvβ3 integrin and CD44 (130). Together, this study demonstrated that OPN can induce a distinct CAF gene set and secretome dependent on receptor engagement.

One mechanism of OPN–fibroblast cross-talk in breast cancer is the engagement of tumor-derived OPN with integrin receptors on mesenchymal stromal cells (MSC), triggering the c-Jun pathway and CCL5 production (131). Under CCL5’s chemotaxis, MSCs consequently acquired CAF markers in an OPN- and CCL5-dependent manner (131). Another mechanism of OPN-dependent CAF activation orbits around TGFβ1, wherein exogenous OPN stimulated TGFβ1 and CAF marker expression in MSCs via the transcription factor myeloid zinc finger 1 (132). Tumor-derived OPN can also bind to αvβ3 integrin and CD44 on fibroblasts to activate Akt, ERK, and Twist1, all contributing to the CAF phenotype (133). Reciprocally, OPN-activated fibroblasts secreted CXCL12 to promote EMT in tumor cells (133). Further *in vitro* data revealed that Tiam1, an Rac exchange factor, negatively regulated OPN and inversely correlated with tumor cell invasion and metastasis in cocultures (134). The same trends were observed in patient datasets (134). Thus, tumor cell–derived OPN harbors the potential to recruit and activate precursor cells into CAFs, serving as a central modulator in the paracrine interactions between epithelial cells and fibroblasts during tumorigenesis. Many solid cancers are susceptible to fibrosis, which can influence tumor progression and therapy response and disrupt adjacent tissue homeostasis, all of which are heavily mediated by tissue-resident fibroblasts and CAFs (123). The multifaceted roles of OPN in this cross-talk warrant further investigation to extract their therapeutic potential.

Beyond breast cancer, head and neck cancer cells secreted high levels of OPN in a dose-dependent manner upon IL6 stimulation from CAFs (135). Reciprocally and in an autocrine manner, head and neck cancer cell–secreted OPN activated the NF-κB pathway through αvβ3 integrin binding to promote tumor progression (135). In colorectal adenocarcinoma, OPN mostly located at the tumor budding and invasion front and colocalized with CAFs and TAMs adjacent to CD44^+^ colorectal adenocarcinoma cells, a defining feature for colorectal adenocarcinoma prognosis (136). Indeed, patients with OPN-positive colorectal adenocarcinoma had significantly shorter OS than OPN-negative patients, suggesting that CAF-derived OPN is likely providing invasion signaling to nearby cancer cells (136).

CAF-derived OPN has also been shown to play a significant role in drug resistance. In HCC, the multikinase inhibitor sorafenib is the standard of care for late-stage patients (137). In nonresponders, OPN emerged as the top upregulated gene signature secreted by CAFs (137). CAF-derived OPN suppressed HCC cells’ sensitivity to tyrosine kinase inhibitors like sorafenib and lenvatinib by binding to adjacent HCC cells, activating a myriad of prosurvival and EMT pathways like RAF, MAPK, PI3K, AKT, mTOR, and PKCα (137). Inhibiting OPN was sufficient to reverse tyrosine kinase inhibitor resistance (137). In addition to resensitizing colorectal adenocarcinoma to tyrosine kinase inhibitors, targeting OPN effectively reduced adult T-cell leukemia tumor cell proliferation in lymph nodes with reduced CAF infiltration (108). Interestingly, anti-OPN did not directly affect leukemia cell growth but rather through CAFs, in which coinoculation of OPN-deficient CAFs and leukemia resulted in a smaller tumor burden and fewer liver metastases (108). Again, these results support that the OPN–CAF axis is a strategic therapeutic vulnerability in many cancers.

Until recently, few studies investigated the roles of CAFs in the context of brain cancer, presumably due to the general lack of fibroblasts in the central nervous system (138, 139). However, using single-cell RNA sequencing, recent analyses revealed a distinct population of “traditional” solid tumor–derived CAFs and their spatial proximity to mesenchymal glioblastoma stem cells (GSC). OPN and CD44 were candidates that emerged from the CAF–GSC interactions, specifically GSC enrichment from neighboring CAFs (138). Additionally, anti-OPN sufficiently reduced neutrosphere formation induced by CAFs (138). These findings suggest that even in solid tumors with intrinsically low CAF infiltration, OPN remains a critical factor that bridges CAF and cancer cell interaction with therapeutic potential (138, 140).

OPN emerged as a central modulator in CAF biology, capable of inducing a distinct CAF phenotype and secretome through receptor-specific signaling while reinforcing its role in the paracrine cross-talk between epithelial cells and fibroblasts during tumorigenesis. This includes EMT, prosurvival signals, ECM remodeling, and targeted therapy resistance, all of which are particularly significant in solid tumor progression. Given the complexity and heterogeneity of CAFs, research on delineating receptor-specific pathways, characterizing CAF subtypes influenced by OPN, and exploring the spatial and temporal dynamics of these interactions across diverse tumor types may be fruitful. Clinically, targeting OPN offers a promising strategy to disrupt fibrosis-driven tumor progression and recondition the TME to enhance the efficacy of existing therapies. OPN may also serve as a biomarker for CAF activity and therapy resistance, providing a dual opportunity for both therapeutic intervention and patient stratification. As solid tumors are often described as “wounds that never heal,” understanding and targeting the OPN–CAF axis could be a key to improving outcomes across a broad spectrum of cancers.

## Endothelial Cells

Throughout the continued evolution of the hallmarks of cancer, sustained angiogenesis continues to hold its place as one of the pathologic features that dictate tumor progression (69, 141, 142). Beyond tumorigenic success, angiogenesis also significantly impacts drug delivery, influencing therapeutic response (143–145). Endothelial cells and the tumor vasculature can be influenced by OPN through cell growth, migration, and VEGF-dependent and VEGF-independent angiogenic cues, with OPN^high^ tumor regions often observed near blood vessels (32, 146). OPN knockout in human endometrial endothelial cells isolated from tumors showed significant impairment in general wound healing activities, including cell proliferation, migration, and invasion (147). Consistently, OPN ablation reduced endometrial tumor burden with decreased CD31 positivity and microvessel density counts, suggesting that tumor-derived OPN exerts significant paracrine effects on endothelial cells to promote angiogenesis (147). Similar observations were found between prostate cancer PC3 cells and endothelial cells. OPN’s binding with integrin receptors on PC3 cells increased ERK1/2 phosphorylation and VEGF expression, which effectively induced human microvascular endothelial cell proliferation and formation of tubule-like structures (148). This phenotype specifically reflects the vessel reorganization stage during angiogenesis, arguing the possibility of a stage-specific effect of OPN on endothelial cells (148).

Overlapping with the MAPK/ERK signaling cascade, PI3K/AKT is often dysregulated in tumors (149, 150). Indeed, OPN secreted by gliomas can induce angiogenic properties in endothelial progenitor cells by activating AKT signaling through αVβ3 integrin binding (151). Of note, their increased migration and tube formation were not in tandem with VEGF, VEGFR1, or VEGFR2 expression but rather with the upregulation of AKT, eNOS, and NO (151). These results suggest that OPN’s role in angiogenesis could be mediated simultaneously through distinct VEGF-independent pathways. Indeed, Dai and colleagues (152) showed that OPN supported endothelial cell survival by dual activation of the ERK and PI3K/AKT pathways, with VEGF serving as a secondary signal. Notably, anti-OPN treatment alone was more potent in inhibiting endothelial cell migration and tube formation than anti-VEGF, supporting OPN as a therapeutic target in angiogenesis (152).

In addition to the MAPK/ERK and PI3K/AKT pathways, OPN secretion by malignant hepatocytes can be driven by inducible YAP^S127A^ (153). Endothelial cells within the liver tumor environment responded to hepatocyte YAP-induced OPN secretion and in turn upregulated their own c-MET expression, reflective of active angiogenesis (153). Furthermore, in human breast cancer cell lines, OPN has been shown to activate Brk/NF-κB/ATF-4 pathways, the cross-talk of which stimulates VEGF to enhance NRP-1–mediated tumor cell motility and vascular endothelial cell growth factor receptor KDR engagement as a bridging point between epithelial and endothelial cells (146).

The essential functions of endothelial cells that are often dysregulated in tumorigenesis can all respond to an increase in environmental OPN availability, including wound healing, migration, invasion, and maintaining adequate microvessel density and angiogenic potential. Notably, these proangiogenic effects of OPN can occur independently of VEGF signaling, positioning OPN as a compelling alternative target for antiangiogenic therapies. This warrants further investigation in preclinical models to evaluate the efficacy of OPN inhibition in disrupting tumor vascularization. Beyond its antiangiogenic potential, modulating OPN–endothelial cell interactions may also offer a strategy to normalize tumor vasculature, thereby enhancing drug delivery and therapeutic response (144). Additionally, the impact of targeting OPN on less-studied endothelial-adjacent stromal populations, such as vascular smooth muscle cells, remains an underexplored area with promising therapeutic implications (154).

## Adipocytes

Obesity and metabolic-related diseases are intimately linked to cancer, generally increasing one’s risk of developing certain malignancies. Of note, the correlation is especially strong for solid tumors that develop in close vicinity to adipose tissues, such as breast, endometrial, and metastatic cancers in the abdominal cavity (155–157). The systemic and chronic inflammation created by obesity is a team effort between adipose tissues and other inflammatory cells. Under normal physiologic conditions, white adipocytes store and brown adipocytes mobilize lipids, altogether maintaining energy stability (155–157). In tumors, adipocytes can adopt alternative functions and influence both cancer and stromal cells, thereby affecting disease progression and treatment outcomes, partly by responding to and secreting OPN (155–157).

The standard experimental procedure to study obesity *in vivo* is the incorporation of a high-fat diet. Mice on a high-fat diet had higher serum OPN, presumably due to obesity-linked systemic inflammation (158). Obese mice also had worse melanoma tumor burden with more adipocyte, macrophage, and osteoclast clustering and an additional increase in OPN (158). These results were not observed in a parallel experimental challenge in OPN-deficient mice, validating the specificity and potency of OPN in tumorigenesis through obesity (158). One mechanism could be the acquisition of brown adipocyte–specific markers (uncoupling protein 1 and PRDM16) with a higher lipid-containing phenotype through PI3K/AKT pathway activation when OPN is overexpressed in adipocytes (159). Indeed, PI3K and AKT inhibition counteracted OPN-driven brown adipogenesis (159). In addition to inflammation, the accumulation of aged visceral adipose tissue was observed to gain p16 senescent marker and OPN positivity (160). The inhibition of OPN decreased senescent adipocyte and macrophage populations and effectively prevented oxidative stress and tissue fibrosis in aged adipose tissue (160). Altogether, these results argue that OPN can shift lipid and energy output in adipocytes, thus influencing tumorigenesis, tissue senescence, and the effectiveness of diet and cold exposure therapy in patients with cancer (159–161).

Matrix proteins such as OPN can mediate the cross-talk between adipocytes and cancer cells. Conditioned media from obese mice–derived adipocytes increased pancreatic cancer cell proliferation and migration compared with that of lean mice (162). In turn, these pancreatic cancer cells stimulated by conditioned media recruited more endothelial cells, reflective of active angiogenesis (162). MMP2 and OPN stood out as the most upregulated cytokines in the conditioned media from obese mice–derived adipocytes, although only OPN knockdown reverted the proliferative, migratory, and angiogenic phenotype in stimulated cancer cells (162). Additionally, OPN and αVβ3 integrin blockades ablated VEGF and HIF1α overexpression driven by OPN (162). On the other hand, periprostatic adipose tissues stimulated with prostate cancer conditioned media saw a 13-fold increase in OPN secretion, suggesting a bidirectional cross-talk between adipocytes and cancer cells mediated by OPN to promote tumorigenesis (163).

## Clinical and Therapeutic Implications

The complexity and extensiveness of OPN’s roles in nearly all cell populations within tumors present a double-edged sword in its clinical introduction. There is an abundance of promising preclinical data on targeting and utilizing OPN as a marker, though major hurdles are foreseeable given its numerous cellular sources and multiaxial roles on tumor and stromal cells. Namely, many ECM-, MMP-, and integrin-based cancer treatments have only seen mild successes in clinical trials because of low bioavailability and severe side effects (164, 165). Nevertheless, the implication of OPN in existing standard-of-care treatments for patients with cancer has been reviewed, in which cancer cell intrinsic and paracrine OPN signaling consistently supports chemotherapy, radiotherapy, and immunotherapy resistance in various cancers (9, 166). Cancer cell–derived OPN conveyed antiapoptotic signaling in lung and breast cancers, whereas mesothelial stromal cell–derived OPN can promote ovarian cancer cell survival and ABC drug effect transporter activity under cisplatin challenge (117, 167, 168). Similar observations were made for radiotherapy resistance, wherein OPN-mediated autophagy shifted radiosensitivity in lung cancer cells (169). Although synergizing anti-OPN treatments with existing immunotherapies awaits clinical investigations, many aforementioned preclinical studies present encouraging results, especially for anti–PD-1 (46, 82, 110).

An increasing number of OPN-centered studies are focusing on examining its systemic distribution and abundance, highlighting its potential as a biomarker (170, 171). Therefore, a parallel understanding of OPN’s systemic kinetics throughout tumorigenesis could distinguish its prospects as a predictor of disease progression and/or metastatic recurrence, which holds a higher likelihood of success for clinical use. Indeed, meta-analyses on more than 200 patient studies revealed that OPN correlated with worse disease-free and relapse-free survival in several cancers, all of which are substantiated by cancer-specific studies (15, 172–174). Furthermore, peripheral OPN levels were also used as a readout marker of chemosensitivity, wherein patients with colorectal cancer treated and responded to cetuximab presented with decreased OPN and increased IL33 (175). The multifaceted role of OPN on epithelial and immune cells extends to numerous pathologic conditions, suggesting that optimizing it as a therapeutic target and as a biomarker could have a clinical impact beyond cancer, such as other inflammatory, autoimmune, and chronic conditions.

Emerging evidence suggests that OPN plays a critical role in modulating lipid metabolism and energy output in adipocytes, thereby influencing tumorigenesis and tissue senescence and the efficacy of metabolic therapies such as dietary interventions and cold exposure in patients with cancer. These findings support a bidirectional cross-talk between adipocytes and cancer cells, mediated by OPN, that promotes tumor progression and systemic metabolic reprogramming. Therefore, OPN should be further researched as a biomarker for systemic inflammation and metabolic dysfunction in cancer across adipose tissue distribution within the body and with aging. This opens new avenues for integrating OPN into therapy response profiling and epidemiologic studies, particularly in relation to body mass index, age, and environmental factors such as climate (176, 177). Future research should explore the mechanisms of OPN’s influence on adipose–cancer cell interactions and assess its potential as a therapeutic target or diagnostic marker in metabolically driven cancers and metastasis (178).

### Conclusion and perspectives

**Figure 2.**
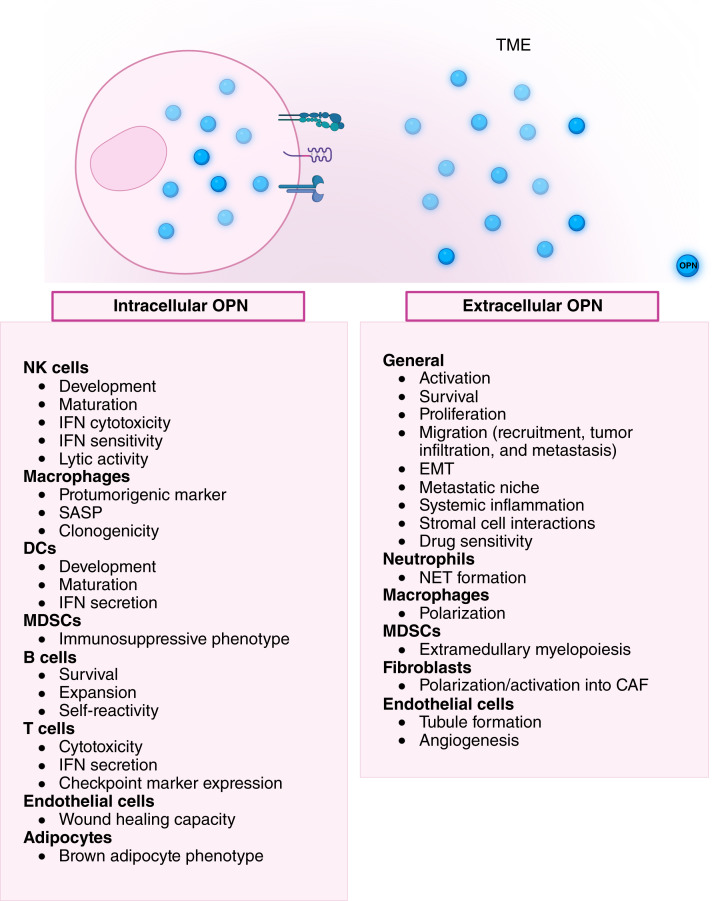
Intracellular and extracellular OPN signaling on tumor stromal cells. Tumor cells and stromal cells can express and retain OPN intracellularly, which supports diverse cell type–specific functions such as promoting macrophage clonogenicity, supporting MDSC immunosuppressive phenotype, maintaining B-cell self-reactivity, sustaining endothelial cell wound healing capacity, and inducing a brown adipocyte phenotype. In parallel, tumor cells and stromal cells can secrete OPN into the TME. Cells expressing OPN receptors respond to extracellular OPN, most of which respond through activation, proliferation, migration, and secondary interactions with other stromal cells. Additionally, extracellular OPN can elicit cell type–specific functions, including NET formation, MDSC extramedullary myelopoiesis, and endothelial cell tubule formation. SASP, senescence-associated secretory phenotype. Created in BioRender. Muller, W. (2025) https://BioRender.com/cf6yfg1.

In the last two decades, research on OPN in the context of cancer has tripled, branching from mechanisms at different stages of tumor progression to the remodeling of the cellular and acellular tumor stroma, therapeutic implications, and biomarker potential. As highlighted in this review, the impact of intracellular and tumor environmental OPN on stromal cell signaling and functions spans the spectrum of pro- and antitumorigenicities (Figs. 1 and 2). As a result, the influence of OPN through stromal cells on the global tumor outcome can vary significantly. Given the extensive posttranslational modifications and cleavage of OPN, its isoform abundance, the source and localization of OPN within a tumor, and its ratio of engagement with different receptors and other binding partners should be considered when evaluating OPN activity and influence on tumor and stromal cells throughout tumorigenesis. In addition to the stromal cell populations discussed in this review, we acknowledge and appreciate that many rare cancers and cancer-specific stromal cell types can be considerably influenced by OPN or secrete unique isoforms of OPN that shift tumorigenesis. Further investigations differentiating OPN’s role on tumor stromal cells in early stage, primary, locally recurrent, and metastatic tumors could provide a multidimensional insight into its protective and antitumoral roles at these critical timepoints. The many studies examining the multiaxial mechanisms and therapeutic implications of OPN in cancer are undeniably pressing invitations for further investigation of its role in improving patient outcomes.
